# Plastic deformation as nature of femtosecond laser writing in YAG crystal

**DOI:** 10.1038/s41598-020-76143-w

**Published:** 2020-11-09

**Authors:** S. S. Fedotov, L. N. Butvina, A. G. Okhrimchuk

**Affiliations:** 1 Mendeleev University of Chemical Technology, 9 Miusskaya Sq, Moscow, Russia; 2 Prokhorov General Physics Institute of Russian Academy of Sciences, Dianov Fiber Optics Research Center, 38 Vavilova Str, Moscow, Russia

**Keywords:** Laser material processing, Mechanical properties

## Abstract

Longitudinal inhomogeneity of tracks inscribed in a YAG crystal and the statistics of nonlinear transmittance of the writing beam is studied under conditions of direct femtosecond laser writing in non-thermal mode. Nonlinear transmittance fluctuations inherent in the laser writing were discovered, and their correlation with track inhomogeneity is established. A model of femtosecond laser writing in crystals is built that includes three modes of plastic deformation in the laser impact zone. The modification threshold of the femtosecond direct laser writing is identified with reaching yield stress.

## Introduction

Direct femtosecond laser writing (DWL) in the bulk of transparent glasses and crystals is a quick and flexible method of forming waveguides and waveguide circuits in a 3D format^1,2^. The method allows one to form spatial distribution of refractive index with a micron resolution by means of a simple coded movement of a tightly focused femtosecond laser beam without any additional technological stages, as opposed to electronic lithography. DWL in crystals is a particularly intriguing technology, because it opens far-reaching perspectives for waveguide formation in any crystals^3,4^. Being a laser medium, the crystal waveguides often have advantages in comparison with optical fibers because they can incorporate large concentrations of rare-earth ions, have better thermal conductivity, larger damage threshold, and have lower impurities causing losses in mid-IR^5^. Waveguides in non-center-symmetric crystals are used in Pockels cells with low control voltage, and as an efficient frequency convertor of low intensity light^6–8^. Recently demonstrated combination DWL and etching open the door to manufacturing of photonic crystal waveguides^9^.

Admittedly, the mechanism and nature of refractive index change in glasses is basically clear. It is connected with the structural rearrangement of the glass grid in a non-thermal modification mode, hence a change in polarization and density^10,11^, or the nanogratings formation^12^, whereas in thermal mode there is an additional aspect to that mechanism—diffusion of ions of which the glass consists^13,14^. Diffusion normally increases the contrast of the refractive index in comparison with a non-thermal mode.

As opposed to glass the mechanism of the refractive index change in crystals suitable for low loss waveguide writing is still not understood, although a broad range of modification regimes was reported for waveguide writing in crystals^3^. The only example of the well-understood nature of refractive index change in crystals is amorphization in quartz^15–17^ and sapphire^18^. Unfortunately, waveguides inscribed in quartz and sapphire under the amorphization mode possess high propagation loss due to crack formation inevitably accompanying the laser writing^15,17,19^.

Ródenas claimed that the cause of the refractive index decrease in ceramic YAG is amorphization too^20^. However, micro-Raman spectra, shown in this paper, allows us to state that the crystal phase is well preserved in the damage area, because Raman linewidths for the damaged crystal region are identical to corresponding linewidths for the pristine crystal. Moreover, the mid-IR reflectance spectra obtained from the modified area in YAG also showed that a crystal lattice survived after modification as opposed to Ródenas’s conclusions^4,21^. Thus, there is no agreement in the scientific community about the nature of DWL in YAG. Forming of voids and phase transformation in sapphire is an example of extreme impact of ultrashort laser pulses on a single crystal, and such mode obviously is not suitable for writing of low loss waveguides^22,23^. Earlier we reported on phase transformation in single crystal YAG, where we wrote tracks in extreme thermal mode, however we haven’t been able to obtain any waveguides in this mode so far^24^.

In cases of low loss waveguides inscribed in crystals, the nature of the refractive index change in the exposed region has not been discovered^25–35^. Meanwhile, knowledge of the state of the matter obtained in the result of moderate modification in crystals in a non-thermal mode is important for discovering ways to improve the uniformity of the modification tracks composing waveguides, as this knowledge opens the way to reduction of scattering loss, which is usually a dominant part in total propagation loss in the depressed cladding waveguides^26,29^.

Here we focused on the study of longitudinal nonuniformity of tracks and the statistics of transmitted pulse energy in the course of track writing in YAG:Nd single crystal and silica glass, and these findings allowed us to draw a conclusion about the nature of modifications in broad ranges of sample translation speeds and laser pulse energies, starting from the modification threshold, which is for the energy range where most smooth tracks are formed suitable for the writing of waveguides with low propagation loss.

## Results

### Statistics of non-linear transmittance

**Figure 1:**
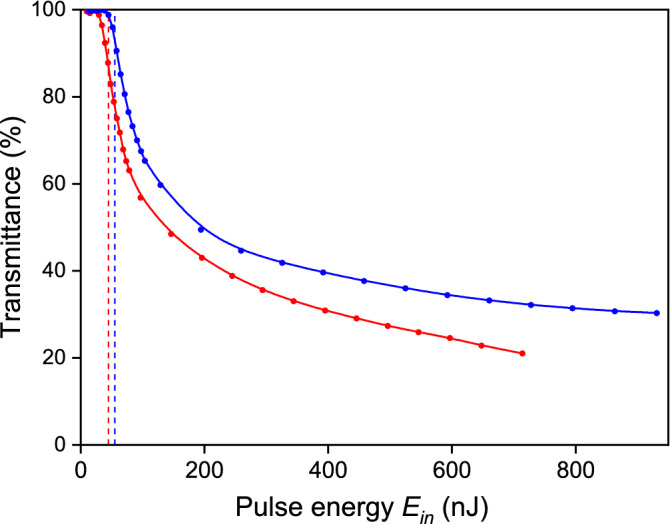
Plot of the non-linear transmittances of YAG: Nd crystal (red) and fused silica (blue) against the energy of the incident laser pulse. The dependences are normalized to linear transmittances of the sample (at low energy, less than 20 nJ). The dashed red and blue vertical lines show the pulse energies at the modification thresholds for the crystal and the glass respectively.

The laser beam was focused through the polished surface a of YAG:Nd(0.3at.%) 10 × 5 × 3 mm plate, which was parallel to the crystallographic plane (211), and the translation direction of the plate was along the [111] crystallographic axis and perpendicular to the [110] crystallographic axis. Dependences of nonlinear transmittances of the YAG:Nd plate and the fused silica plate on the laser pulse energy *E*_*in*_ were obtained under the condition where each laser pulse was focused to a new, unmodified place in the plate in order to avoid the memory effect^36^ (Fig. 1). These dependencies in general repeat the ones previously obtained^36,37^. The following study of the samples with an optical microscope, operating in the transmittance mode, revealed that the pulse with energies of 40 nJ and 50 nJ does not produce any modification in the crystal and glass correspondingly. Thus, we estimated that the threshold modification energy is equalled to 45 nJ for the crystal, and 55 nJ for glass. The nonlinear transmittance of the YAG:Nd crystal equals 87% at the threshold, that is, 13% of the laser pulse energy passing the crystal—air interface is absorbed by the plasma in assumption that plasma reflection is negligible. The transmittance of the silica glass is equalled to 91% at the modification threshold energy.

To analyse the statistics of the nonlinear transmittance, it appeared to be more convenient to present the experimental dependences on the number of laser pulses that overlap with each other in the modification region than on the translation speed^3^:

1 $$P=\frac{D{f}_{rep}}{{V}_{sc}}$$

where *D* is the diameter of the laser beam waist, *f*_*rep*_ is the laser pulse repetition rate. Below we will call this parameter as the overlap *P*.

**Figure 2:**
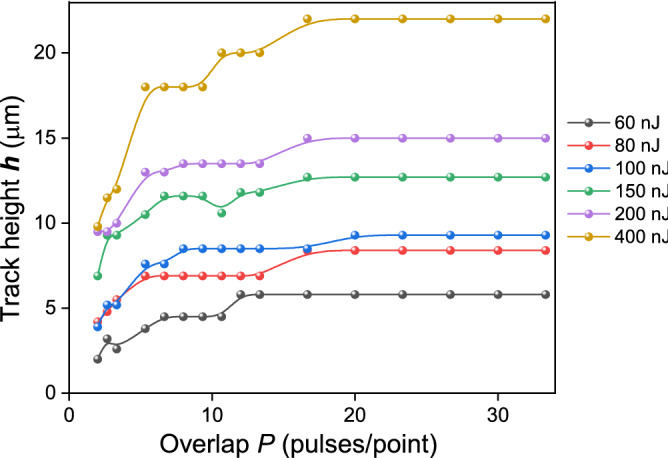
Plot of the track height *h* against the overlap *P* while writing tracks with a constant speed *V*_*sc*_ in the range 0.03–0.8 mm/s. *f*_*rep*_ = 1 kHz and the pulse energies *E*_*in*_ shown in the plot.

The inscribed tracks of reduced refractive index in YAG:Nd crystal had a traditional cross section shape, strongly elongated along the beam^20^. The transverse size of the track along the beam will be referred to as the track height *h*. Figure 2 shows the plot of the track height *h* in the YAG:Nd crystal against the overlap *P* for different laser pulse energies *E*_*in*_.

**Figure 3:**
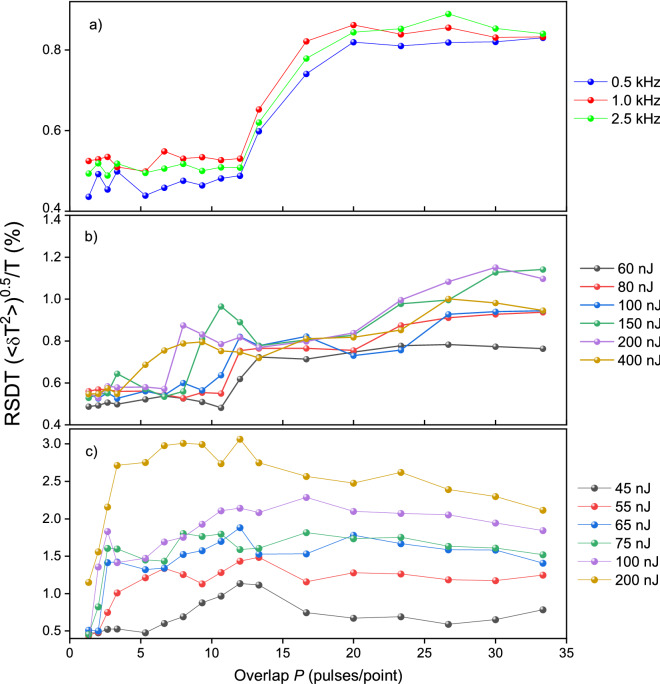
Plots of the RSDT of YAG: Nd crystal (**a**,**b**) and silica glass (**c**) against the overlap *P* at different laser pulse repetition rates and constant laser pulse energy incident on the sample *E*_*in*_ = 70 nJ (**a**), and different laser pulse energies *E*_*in*_ and constant rep rate equal to 1 kHz (**b**,**c**).

Figure 3 shows the relative standard deviation of the transmittance RSDT= $\sqrt{\langle \delta {T}^{2}\rangle }/T$ obtained by statistical processing of the nonlinear transmittance measurements according to formula (3) (“Methods”). It was found that for YAG:Nd RSDT is about 0.5% for overlaps less than 10, and for the pulse energy not exceeding 100 nJ (Fig. 3b). The surge of RSDT from 0.5 to 0.7% is observed under the overlap of 11–15 pulses, and then its growth significantly slows down and remains in the range of 0.7–1.0%. The surge of RSDT shifts toward smaller overlaps when the pulse energy increases over 100 nJ.

We were convinced that this surge is not associated with instabilities of the translation stage at low speeds. To establish this, experiments were carried out at three different pulse repetition frequencies. If the surge was related to the translation stage speed *V*_*sc*_, it would appear at different overlaps *P* depending on the velocity *V*_*sc*_ (see formula (1)). However, it has been found that the surge always appears at the same overlaps within the measurement error (Fig. 3a). Therefore, we believe that the observed RSDT surge has a fundamental nature and is due to the peculiarities of the femtosecond laser modification in crystals. Analogous experiments were done with silica glass in order to compare statistics of non-linear transmittance in a crystal and glass. No surges of RSDT were observed for glass (Fig. 3c).

### Longitudinal inhomogeneity of tracks

**Figure 4:**
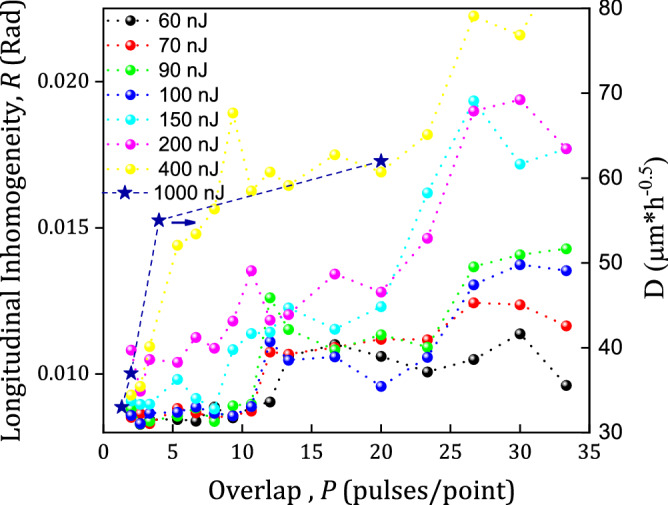
Plot of the longitudinal inhomogeneity *R* (circles) and etching rate *D* (stars) of tracks in YAG:Nd crystal against the overlap. The energies *E*_*in*_ of laser pulses incident on the sample are shown on the graph.

Longitudinal inhomogeneity of the tracks in YAG:Nd was investigated with Fourier analysis of their phase images (Method). The plots of the track longitudinal inhomogeneity *R* against the overlap *P* are shown in Fig. 4.

**Figure 5:**
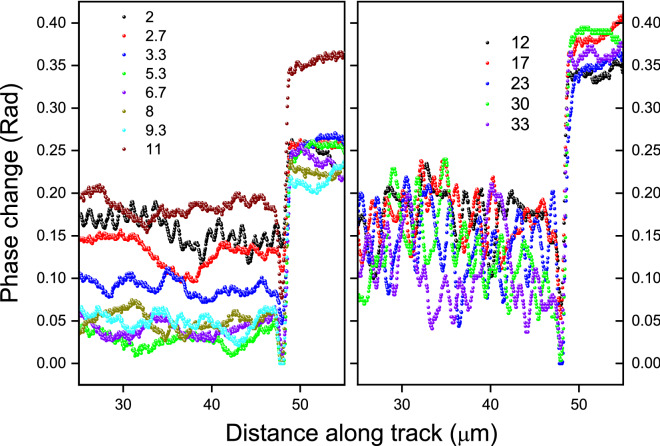
Optical phase profiles along the tracks in the YAG:Nd crystal with different overlaps *P* shown on the graph. Laser pulse energy *E*_*in*_ = 70 nJ.

In order to understand the dynamics of writing we have investigated the ending of a mm-long track written in YAG:Nd under the condition where the laser was switched on while the beam waist was scanning inside the crystal at constant speed. The optical phase profiles along the central lines of tracks under view along the direction of the laser beam are shown in Fig. 5. On the graph the increase of the distance coincides with the direction of writing, which finishes at the 48 μm point. The end of the writing is marked by a characteristic dip for all tracks. Random phase fluctuations along the tracks inscribed with overlaps *P* = 2.7–11 do not differ in amplitude and characteristic spatial frequencies from those in unirradiated crystal regions. The increased phase fluctuations along the track with *P* = 2 are obviously caused by a purely geometric factor due to a very small overlap, which is not relevant to our study. In tracks inscribed with overlaps *P* > 12 both the amplitude and the frequencies of random phase oscillations are significantly increased compared to pristine crystal regions.

**Figure 6:**
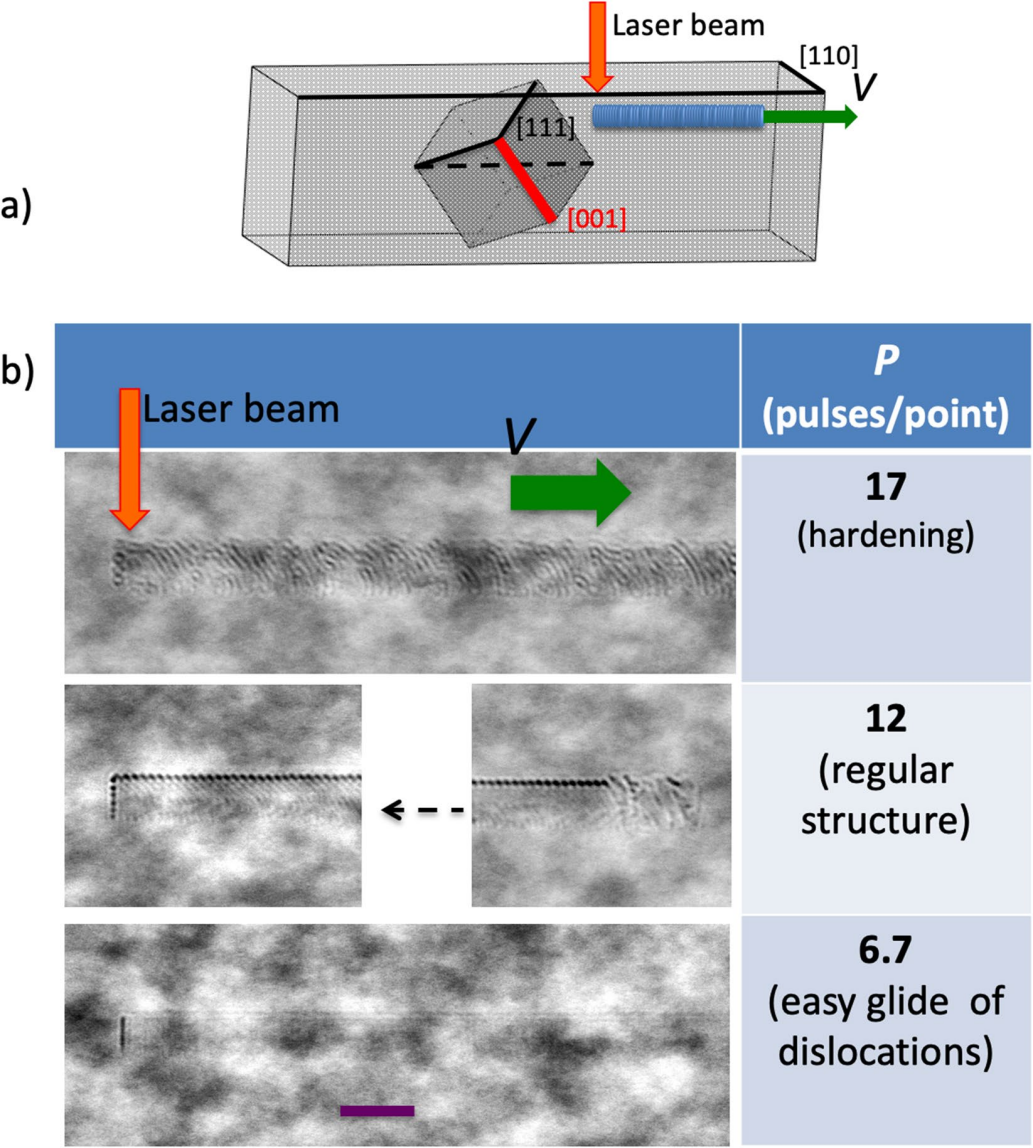
(**a**) Scheme of DLW with the writing direction relative to crystallographic axes along with elementary crystallographic cell shown inside the sample. (**b**) Representative side phase images (under view along [110]) of tracks inscribed at different regimes of plastic deformation. The direction of the tracks is along [111] crystallographic axis; the direction of the laser beam is along [211]. The plane of the figure is a (110) crystallographic plane. The writing direction is from right to left. E_in_ = 70 nJ. Scale bar is 10 μm.

Figure 6 shows phase images of the tracks obtained under the view perpendicular to the laser beam. For small overlaps *P* = 3–7, the phase incursion is smooth and uniform along the track, and hardly visible on the background of random phase fluctuations of apparatus and pristine crystal. A regular phase modulation in the form of oblique stripes is observed along the tracks with overlaps *P* = 9–13, while the direction of the stripes coincides with the direction of the crystallographic axis [100]. The period of the regular modulation lies in the range of 1.19 – 1.27 μm. Chaos is observed in the phase images of the tracks with a large overlap of 17 or greater.

While writing tracks, we recorded videos under top view in the direction of the laser beam. Two representative videos of the writing under small and large overlaps (P = 3.3 and 23) are in the Supplementary Videos1 and 2 correspondingly. In the videos one can see stable writing with the small overlap and unstable chaotic modification in front of the track being written with a large overlap.

## Discussion

The femtosecond modification of a transparent dielectric begins with the generation of electron plasma^2,38^. Next, the conversion of the electron plasma energy into the energy of the crystal lattice occurs, with a small fraction of the energy being emitted by the plasma. We believe that the modification of the YAG:Nd crystal under the impact of femtosecond pulses, i.e., constant changes in the crystal lattice, is plastic deformation accompanied by a generation of vacancies, their fusion into vacancy disks, and the subsequent generation of dislocations on them. Generation of slip dislocations is a necessary condition for plastic deformation^39,40^. A decrease in the refractive index in the modification region is the result of the crystal density decrease during the plastic tension under the impact of internal pressure arising in the region of the electron plasma, and under conditions of comprehensive compression from the side of the surrounding unirradiated crystal lattice. While writing a track, the plasma region moves throughout the crystal from pulse-to-pulse. The crystal was subjected to the highest tension at the plasma region, and the tension partially relaxed in the track outside the plasma region. Thus, the end of a track, where the last laser pulses impacted, is subjected to highest tension. This is reflected in the phase profile along the track. The dip at the end of writing corresponds to the region with the highest tension, and the dip amplitude increases with the overlap *P* (Fig. 5).

The decrease in crystal density is associated with the generation of vacancy disks and agglomeration of dislocations. The dislocation formation at the focal point of the laser beam was revealed with transmission electron microscopy under exposure to femtosecond pulses at 800 nm in single crystal MgO^41^. However, it should be noted that the pulse fluence used in this work was nearly 100 times higher than in our experiments, and this has also led to the generation of dislocation far from the exposed region^42^.

Next, we show that at the modification threshold the generated pressure fairly matches YAG yield stress. The events associated with the deformation of the crystal in the region of modification finish on the time interval of 10 ns^42–44^. Heat transfer from the pulse absorption zone can be neglected since the characteristic time of this process $\tau ={c}_{p}\rho {d}_{s}^{2}/\kappa$(*c*_*p*_ is the specific heat, *ρ* is the density, *d*_*s*_ is the characteristic size of the plasma region, *κ* is temperature diffusivity) exceeds 1 μs even for the smallest *d*_*s*_ ≈ 6 μm. Immediately after the pulse passage (*t*≈0.5 ps) the crystal lattice has not heated up from the absorbed energy yet, and the Gibbs free energy *Φ* of the electron plasma region has increased by the absorbed pulse energy $\Phi =d({E}_{a}-TS)\simeq {E}_{a}$ . Here we neglected the second term in brackets because of the proximity of the temperature to zero and the smallness of entropy^45^. On the other hand, in accordance with the basic thermodynamic relation $d\Phi =-SdT+{V}_{0}dp\simeq {V}_{0}{p}_{0}$, where *V*_*0*_ is the volume occupied by the electron plasma, *p*_*0*_ is the excessive pressure in the electron plasma region. Thus, we obtain the formula for pressure:

2 $${p}_{0}=\frac{{E}_{a}}{{V}_{0}}$$

A single-pulse modification of a YAG crystal occurs when at least 45 nJ·0.13 = 5.9 nJ is absorbed (at threshold modification energy). According to Fig. 3, the height of the plasma cloud at the energy threshold of modification does not exceed 6 μm, therefore ${V}_{0}=\frac{\pi }{4}{D}^{2}h=4.7$ μm^3^. In accordance with formula (2), an average pressure of 5.9 nJ/4.7 μm^3^ = 1.3 GPa is created in the plasma region. However, the absorbed energy density is extremely inhomogeneous in the volume of the plasma, especially along the direction of the laser beam propagation, as follows from numerical calculations made for silica glass and which is qualitatively applicable to the YAG crystal^46^. Thus, it should be expected that the local energy density manyfold exceeds the average value. Accordingly, the same behaviour is expected for pressure. Thus 1.3 GPa is an estimation of the low limit for pressure developed in the plasma region.

When studying the synthesis of YAG nanoceramics^47^, and the deformation of nanoceramics^48^, it was found that a pressure of 4 GP is required to activate plastic deformation of YAG microcrystals at a temperature less than 400 °C (yield stress). The activation pressure decreases to 2 GPa with the temperature increasing up to 1500 °C. Based on the arguments of the previous section, we believe that such pressure locally arises within the plasma zone, and it initiates plastic deformation of the crystal.

In good approximation, sizes of the plasma cloud do not depend on the overlap *P* due to the small changes in the linear and nonlinear refractive indexes and multiphoton absorption coefficient. However, the modification region size *h* depends on overlap *P* (Fig. 2). We consider that the first laser pulse plastically deforms a small inner part of the plasma region, in which the shear stress is maximal, and hence the yield strength is firstly reached here. The height of this deformed region is about 2 μm at the threshold pulse energy, and about 10 μm at the pulse energy of 400 nJ (Fig. 2). Excess of the laser pulse energy above the threshold does not lead to increased pressure, since the yield stress has already been reached, and the "excess" energy is spent on the generation of new point defects and dislocations. Accordingly, the Gibbs free energy *Φ* does not increase due to an increase in entropy, which is included in the second term in the equation $d\Phi =d({E}_{a}-TS)$. In the unmodified part of the plasma region, where plastic deformation does not start yet, the dislocation concentration increases from pulse to pulse due to combination of emerging vacancies and dislocation intergrowth from the modified region. These processes lead to the yield stress decrease in these regions. Dislocation accumulation effect increases with the overlap increase. Thus, the region of plastic deformation expands with an increase in the overlap from 3 to 15–20 due to a decrease in the yield stress. This expansion takes place in the direction of the crystal regions exposed to the electron plasma, and where therefore the free energy *Φ* is accumulated and can be spent for plastic deformation. Thus, the region of plastic deformation expands along the beam. The model explains the increase in track height *h* with an increase in overlap *P* from 3 to 20 (Fig. 2). The expansion stops at the boundary of the plasma region, which manifests itself in the saturation of the track height. The plasma cloud height increases with the pulse energy increase, so the saturation level of the track height accordingly depends on the pulse energy.

During the sequential action of laser pulses on the same crystal region an easy glide mode of the plastic deformation stops when the motion of the dislocations stops by itself. This phenomenon, well-known in the deformation of metals, macroscopically manifests itself as a hardening mode of the plastic deformation^49^. The hardening occurs with an overlap of more than 15 pulses (Fig. 6). A hardened region of the crystal reacts elasto-plastically to subsequent pulses. It is significant that in the hardening mode, in contrast to the easy glide plastic deformation, stresses are accumulated from pulse to pulse, and than stochastically relaxed with generation of nano/micro pores, which manifest itself in inhomogeneity in the phase image (Fig. 6).

This reasoning is supported by videos of the track writing under small and large overlaps. Under small overlap (*P* = 3.3, Video 1) smooth and stable writing is observed, for which modification is completely within the exposed area of the crystal. Under large overlap (*P* = 23, Video 2) we see chaotic deformation even of the unexposed region of the crystal in the direction of the beam waist movement, indicating chaotic expansion of the hardened crystal under the impact of laser pulses.

The regular structure that looks like stripes nearly parallel to the [100] crystallographic axis at overlaps *P* = 9–13 precedes the regime of complete hardening (Fig. 6). Earlier we observed a similar self-organized structure with similar pitch of 0.91 μm in YAG:Cr^4+^ while inscribing tracks at wavelength of 400 nm^4^. Stripes were perpendicular to the track, but again parallel to [100]. The regular structure has a common nature neither with the laser induced periodic surface structure (LIPSS) observed in YAG^50^, nor with volume nano-gratings observed in fused silica^12^. These statements are supported by experimental facts: (1) orientation of the stripes follows crystallographic axes and is not related to polarization; (2) the structure is not formed under high overlap of laser pulses. We believe that we are dealing with self-organization deformation processes that are intrinsic to crystals, and the laser beam plays the role of an initiator only. When the dislocation density reaches some critical value, a track with homogeneous dislocation distribution becomes an unstable dissipative system^51^. Periodical agglomeration of dislocation reduces system energy and makes it stable^52,53^. A sharp spontaneous transition from the chaotic agglomeration of dislocation to the regular structure happens with some delay after the beginning of track inscription, and it can be considered as a bifurcation point^51^. The amplitude of delay is obviously connected with dislocation mobility, which is reduced with the overlap.

Surges are observed in both dependences of the longitudinal inhomogeneity *R* and RSDT on the overlap at the same range overlaps of 11–15 (Fig. 3). Thus, a correlation has been found between fluctuations of the nonlinear transmittance during the track writing and the longitudinal inhomogeneity of the track *R*. Therefore, RSDT can serve as an in situ indicator of the inscribing track quality during laser writing. It can be expected that the most stable writing of smooth tracks should be under pulse energies that exceed the threshold energy by no more than 2–2.5 times, which is in the range of 45 nJ–100 nJ, and under the overlap range of 3–7 pulses/point.

We connect the surge in the dependences of RSDT and the longitudinal inhomogeneity *R* on the overlap *P* with the hardening (Figs. 3, 4). In the hardening mode (*P* > 15, Fig. 6) the concentration of dislocation reaches a critical value, when they spontaneously gather in nano/micro pores, the distribution of which are therefore extremely heterogeneous, and subsequently causes the track inhomogeneity. Since the beam propagates through the forming nano/micro pores at a large overlap of pulses, it experiences unequal focusing from pulse to pulse, which in turn causes fluctuations in the nonlinear transmittance.

Recently a new disruptive technology of a photonic crystal waveguide in YAG crystals has been proposed^9,54,55^. It is based on the phenomenon of a dramatic etching rate increase in the tracks inscribed by a femtosecond laser, however the theory of this phenomenon is not developed yet^56^. We believe that the etching rate increase is due to an anomalous multiplication of dislocations leading to their agglomeration accompanied by formation of nanocracks. The dependence of the etching rate upon pulse energy and writing velocity was measured under writing conditions similar to our study^54^. These results allow us to build a dependence of etching rate (parameter D) upon overlap P (Fig. 4). One can see good correlation of the etching rate plot with the plot for roughness (for the highest energy) indicating a common origin of the dependences. This finding supports the statement that plastic deformation is a basic phenomenon lying in the nanostructuring in crystals.

It is important that no surges are observed in a similar dependence of RSDT for silica glass. Instead there is an even decrease in RSDT at large overlaps. The qualitatively different dependence for YAG and silica glass is explained by a fundamentally different nature of plastic deformation in crystals and glasses. In crystals, it is the generation and slipping of dislocations that have long-range fields. In glasses, rearrangement of the short-range order is only possible; therefore, hardening in glasses is not observed. In this regard, it is not surprising that no surge in the graphs of RSDT is observed for silica glass (Fig. 3c).

## Conclusions

The nature of direct femtosecond laser writing in YAG crystal in the non-thermal mode lies in plastic deformation on a microscale. The decrease of the refractive index in the modification area is caused by dislocation and vacancy agglomeration that happen when its concentration reaches a critical value. We have identified three modes of plastic deformation relevant to direct laser writing. The first mode is characterized by an easy glide of dislocation, the second is the hardening mode, and the third is an intermediate mode with self-organization in the dislocation system. The mode of plastic deformation is defined by two independent parameters: laser pulse energy and overlap of exposed areas by laser pulse sequences.

Our conclusion on the nature of femtosecond laser writing is based on analysis of the statistics of non-linear transmittance of crystal YAG and silica glass and also of the longitudinal inhomogeneity of tracks of the refractive index change. Accidental fluctuations of nonlinear transmittance of fundamental origins have been found during track writing both in crystal and glass. However, a substantially different dependence of root-mean-square deviation of nonlinear transmittance on overlap of exposed areas by laser pulse sequences has been found for these materials.

We presume that the surge in the dependence of the longitudinal inhomogeneity and the transmittance noise on the overlap in the crystal is connected with the change from the plastic deformation mode with an easy glide of dislocations to the hardening mode. In the transitory mode under the overlap of 9–13, the modulation of the refractive index has been observed along the track with a period of approximately 1.2 μm, which we believe is pertaining to self-organization in the dissipative dislocation system. The plastic deformation mode with an easy glide of dislocations has to be provided for direct laser writing of waveguides with a low scattering loss, which takes place under small overlap of laser pulses (P = 3–7) unlike the common view on direct laser writing in crystals which implies that the smooth and homogeneous optical properties along the waveguide can be obtained under a large overlap.

Humankind has been taking advantage of plastic deformation since the dawn of time, for example in metal shaping. However, in our research we are possibly the first to describe plastic deformation initiated not on the surface of a crystal, which usually happens when a sample is exposed to mechanical pressure, but inside it.

We expect that the proposed model of laser writing can be expanded on other crystals and will create a new approach in the study of direct femtosecond laser writing in crystals. We believe that plastic deformation is the basic phenomenon underlying the dramatically increased etching rate of YAG crystal exposed by ultrashort pulses, and could play a key role in the development of nanolithography for crystals^9,54^.

## Methods

### Non-linear transmittance

The statistics of nonlinear transmittance of femtosecond laser pulses of YAG:Nd (0.3 at%) and silica glass plates in the course of writing tracks of a modified refractive index in them was studied using a commonly used transverse scheme for direct laser writing of waveguides^1^. The femtosecond laser beam at a wavelength of 1030 nm was focused by a 0.65 numerical aperture objective lens under a polished surface of a 3 mm thick plate at a depth of 50 μm. The plate was mounted on a precision 3D translation stage (Aerotech) and translated parallel to the polished surface at a constant speed *V*_*sc*_ in the direction perpendicular to the beam. The plane of the laser beam polarization was perpendicular to the translation. The calculated beam waist diameter *D* was 1.0 μm. The pulse duration was 180 fs, and the repetition rate did not exceed 2.5 kHz, and was equal to 1 kHz in most of the experiments. Such low repetition rate allowed for avoiding effects with heat accumulation from pulse to pulse. The energy of the laser pulse incident on the sample *E*_*in*_ was controlled using a polarization attenuator, while 30% of the beam power was diverted by the beam splitter to the energy meter head (PD10-C, Ophir). In addition to the standard direct laser writing setup, the beam that passed through the sample was collected on a second energy meter head with an objective lens having a numerical aperture of 0.75. Thus, the energies of each pulse incident on the sample and transmitted through it were simultaneously measured, and subsequently the statistics of the non-linear transmittance were analysed.

Fluctuations in the non-linear transmittance have three contributions of different natures. Aside from the stochastic part of absorption/reflection of the electron plasma, the noises of the laser itself and the pulse energy detector contribute to these fluctuations. In Appendix 1, a formula is derived for the relative standard deviation of the transmittance (RSDT):

3 $$\frac{\sqrt{<\delta {T}^{2}>}}{T}=\sqrt{\frac{<\delta {E}_{A}^{2}>}{{E}_{A}^{2}}-\frac{<\delta {E}_{detA}^{2}>}{{E}_{A}^{2}}-\frac{<\delta {E}_{in}^{2}>}{{E}_{in}^{2}}}$$

### Longitudinal inhomogeneity of tracks

A stack of microscopic images containing a 110 μm-long section of a track inscribed in the YAG:Nd crystal and captured along the direction of the writing laser beam was analysed using Quantitative Phase Microscopy (QPm)^57^. Image capture was done on optical microscope Olympus BX-61 equipped with camera Retiga 3000 and an objective lens Olympus UPlanFLN-40x (NA = 0.75)*.* The sample was back illuminated by green light from a halogen lamp passing through an interference filter centred at 503 nm with bandwidth of 10 nm. An image focused on a track and two images defocused by ± 1 μm were captured using computer-controlled z-stage with a piezo actuator. The two-dimensional phase incursion distribution was computed from above three images with QPm software (Iatia Ltd.) and was obtained for each pair of the laser pulse energy *E*_*in*_ and the overlap *P* (Fig. 3b).

**Figure 7:**
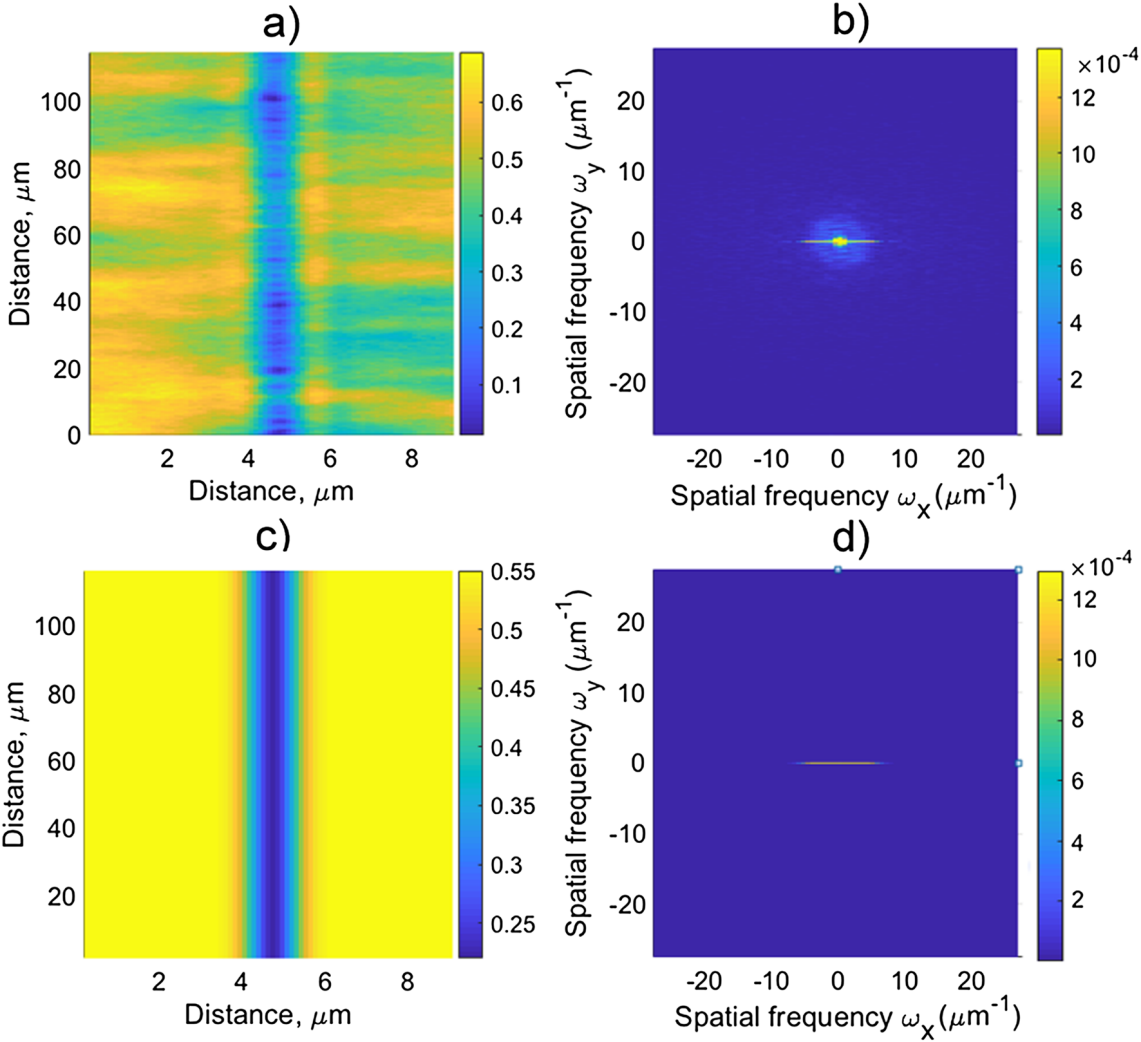
The phase image of the track written in YAG (**a**), and its DFT module (**b**). *P* = 17, *E*_*in*_ = 70 nJ. The phase image of the ideal model track with a Gaussian transverse phase profile and a width of 1 μm (**c**), and its DFT module taken with the Hamming weight function (**d**). The upper phase limit in the Fourier images is set 10^4^ times lower than the maximum phase excursion in the track in order to make the noise contribution visible.

A phase image underwent a two-dimensional discrete Fourier transform (DFT) with the Hamming weight function. For example, a section of the track inscribed with *P* = 17 and *E*_*in*_ = 80 nJ, and its Fourier transformation is shown in Fig. 7a,b, respectively. Figure 7d shows DFT taken from an ideal model track (Fig. 7c) with a Gaussian phase profile, having amplitude of 0.1 Rad and nearly the same width of 1 μm as a real track. DFT of a real track is separated to a “regular “ part, which is the same as for the ideal model track (Fig. 7c), and a “noise” part, which predominantly lies in the upper and lower spatial frequency regions *ω*_*y*_ > *ω*_*1*_ and *ω*_*y*_ < *ω*_*1*_ (symmetrical to each other), where *ω*_*1*_ is some cut-off frequency. Therefore, the formula expressing the root-mean square phase inhomogeneity across the track reads:

4 $$R=\frac{2}{NM}\sqrt{\sum_{n=1}^{N/2}\sum_{m=H}^{M/2}{\left|{DFT(\varphi )}_{n,m}\right|}^{2}}$$

where *N* and *M* is the number of pixels in this image by abscissa (across a track) and ordinate (along the track) correspondingly, $H={\omega }_{1}{L}_{y}/\pi$. The rigorous mathematical justification of the formula (4) is given in Appendix 2.

## Supplementary information

Supplementary Video Legends.

Supplementary Appendixes.

Supplementary Video 1.

Supplementary Video 2.
