# Supplementary material for: Plastic deformation as nature of femtosecond laser writing in YAG crystal
Source: Sci Rep. 2020 Nov 9;10:19385. doi: 10.1038/s41598-020-76143-w (PMC7653935; doi:10.1038/s41598-020-76143-w)
Supplement: Supplementary file 1 — Supplementary Video Legends. [file 41598_2020_76143_MOESM1_ESM.pdf]

Video 1. Inscription of a track under overlap P=3.3. The view is in the laser beam direction, while a halogen lamp backlights a track in the opposite direction.

Video 2. Inscription of a track under overlap P=23. The view is in the laser beam direction, while a halogen lamp backlights a track in the opposite direction.
