# Supplementary material for: Plastic deformation as nature of femtosecond laser writing in YAG crystal
Source: Sci Rep. 2020 Nov 9;10:19385. doi: 10.1038/s41598-020-76143-w (PMC7653935; doi:10.1038/s41598-020-76143-w)
Supplement: Supplementary file 2 — Supplementary Appendixes. [file 41598_2020_76143_MOESM2_ESM.pdf]

Plastic Deformation as Nature of Femtosecond Laser Writing in YAG crystal

S.S. Fedotov[1], L.N. Butvina[2], A.G. Okhrimchuk[1,2*]

[1]Mendeleev University of Chemical Technology of Russia, International Center of Laser Technology, 9 Miusskaya Sq., Moscow, Russia.
[2]Prokhorov General Physics Institute of Russian Academy of Sciences, Dianov Fiber Optics Research Center, 38 Vavilova Str., Moscow, Russia.

E-mail: okhrim@fo.gpi.ru

Appendix 1.

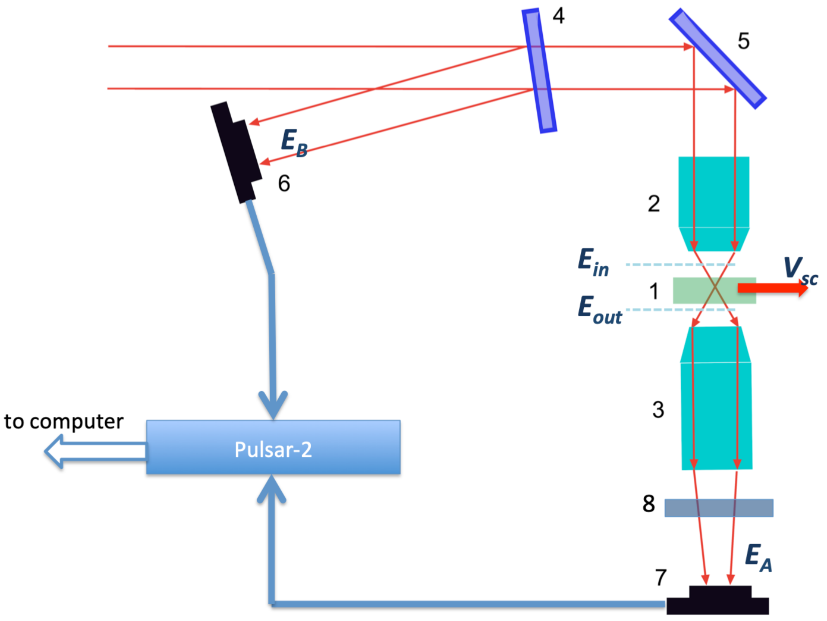

Fig.S1. Optical setup for measurement of the nonlinear transmittance. 1 is a sample, 2 and 3 are the focusing and the collecting objective lenses, 4 is the beam splitter, 5 is the HR mirror, 6 and 7 are the energy meter heads PD10-C (Ophir), 8 is the dispersive silica plate.

The nonlinear transmittance of a sample is expressed by the formula:

$$T(E_{in}) = \frac{E_{out}(E_{in})}{E_{in}} \qquad \text{(S1)},$$

where E_{in} and E_{out} is the mean value of the laser pulse energy, entering and going out of the sample correspondingly (here we omit the Fresnel reflection for simplicity). E_{in} and E_{out} are linearly connected with energies, measured by the energy meter heads: $E_{in} = \eta_1 E_B, E_{out} = \eta_2 E_A$, where η_1 and η_2 are apparatus coefficients.

Let us consider deviation of transmittance δT, caused by a fluctuation of the non-linear absorption coefficient. Such fluctuation could have happened for several reasons: change of the beam waist diameter, caused by perturbations in the media; fundamental non-reproducibility from pulse-to-pulse of (1) electron concentration or (2) sizes of plasma cloud, and (3) deviation of laser pulse energy δE_{in}, caused by instability of the laser. Then we can write a formula for energy of a pulse, going out the sample E_{out1}:

$$E_{out1} = (E_{in} + \delta E_{in})(T + \delta T) \qquad \text{(S2).}$$

This energy can be only measured together with deviation δE_{detA}, caused by detector noise:

$$\frac{E_{out1}}{\eta_2} + \delta E_{detA} = E_A + \delta E_A \qquad \text{(S3).}$$

Where δE_A is the energy deviation from the mean value E_A, caused by all kind of fluctuations and noises, in contrary to δE_{detA} that is caused by the detector noise only. Then putting (S2) in (S3), and neglecting by the term of the second order of smallness $\delta E_{in}\delta T$ under assumption $\delta T \ll T, \delta E \ll E$, we get:

$$E_A + \delta E_A \cong \frac{E_{in}T + E_{in}\delta T + T\delta E_{in}}{\eta_2} + \delta E_{detA} \qquad \text{(S4).}$$

Then we raise to the square both parts of the equality (S4), and average each of them under assumption that the stochastic processes $\delta E_{in}, \delta T$ и δE_{detA} are absolutely uncorrelated ($< \delta E_{in}\delta E_{detA} >= 0$ and so on):

$$E_A^2 + < \delta E_A^2 > \cong \frac{E_{in}^2 T^2 + E_{in}^2 <\delta T^2> + <\delta E_{in}^2> T^2}{\eta_2^2} + < \delta E_{detA}^2 > \qquad \text{(S5).}$$

So as $E_A^2 = \frac{T^2 E_{in}^2}{\eta_2^2}$, $E_{in}^2 = \frac{E_A^2 \eta_2^2}{T^2}$ and $\frac{T^2}{\eta_2^2} = \frac{E_A^2}{E_{in}^2}$ equality (S5) is transformed to:

$$\frac{\sqrt{<\delta T^2>}}{T} = \sqrt{\frac{<\delta E_A^2>}{E_A^2} - \frac{<\delta E_{detA}^2>}{E_A^2} - \frac{<\delta E_{in}^2>}{E_{in}^2}} \qquad \text{(S6),}$$

where $\sqrt{< \delta T^2 >}$ is RMS deviation of the non-linear transmittance, caused by a fluctuation of the non-linear absorption coefficient.

In the absence of the sample $T \equiv 100\%$, and $\sqrt{<\delta T^2>} = 0$ so we get from (S6):

$$\frac{\sqrt{<\delta E_A^2>}}{E_A} = \sqrt{\frac{<\delta E_{detA}^2>}{E_A^2} + \frac{<\delta E_{in}^2>}{E_{in}^2}} \qquad \text{(S7).}$$

Equation (S7) allows to estimate contributions of detector and laser noises $<\delta E_{detA}^2>$ and $<\delta E_{in}^2>/E_{in}^2$. So as the laser pulse energy was controlled by a passive polarization attenuator, the relative laser noise $<\delta E_{in}^2>/E_{in}^2 = const$. We used the 20 nJ measurement limit of the energy meter head for all series of the experiment, and in order to fit E_A deviation range to this measurement limit we attenuated a pulse coming out from the objective lens 3 with the dispersive silica plate 8 (Fig.S1). The absolute detector noise $<\delta E_{detA}^2>$ for a single measuring limit is constant too. Thus function of $\sqrt{<\delta E_A^2>}/E_A$ on the E_A in accordance to (S7) has the form:

$$f(x) = \sqrt{C_1^2 + (C_2/x)^2} \qquad \text{(S8),}$$

where $C_1 = \sqrt{<\delta E_{in}^2>/E_{in}^2}$, and $C_2 = \sqrt{<\delta E_{detA}^2>}$. Experimental dependence of $\sqrt{<\delta E_A^2>}/E_A$ upon E_A, measured with absence of a sample (Fig.S2), was approximated by formula (S8), and we have: $\sqrt{<\delta E_{in}^2>/E_{in}^2} = 0.59\%$, $\sqrt{<\delta E_{detA}^2>} = 47\ pJ$ (for the 20 nJ measurement limit of the meter head PD10-C).

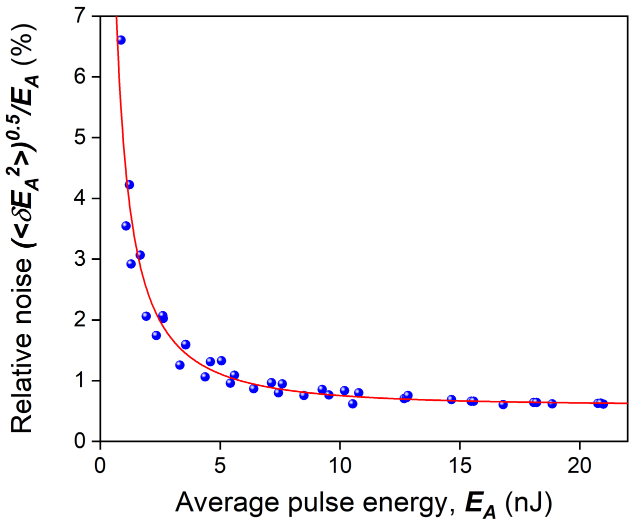

Fig.S2. Plot of relative RMS deviation of the pulse energy, measured by the energy meter head A, against the average energy (points). The solid red line is an approximation by formula (S8).

Appendix 2

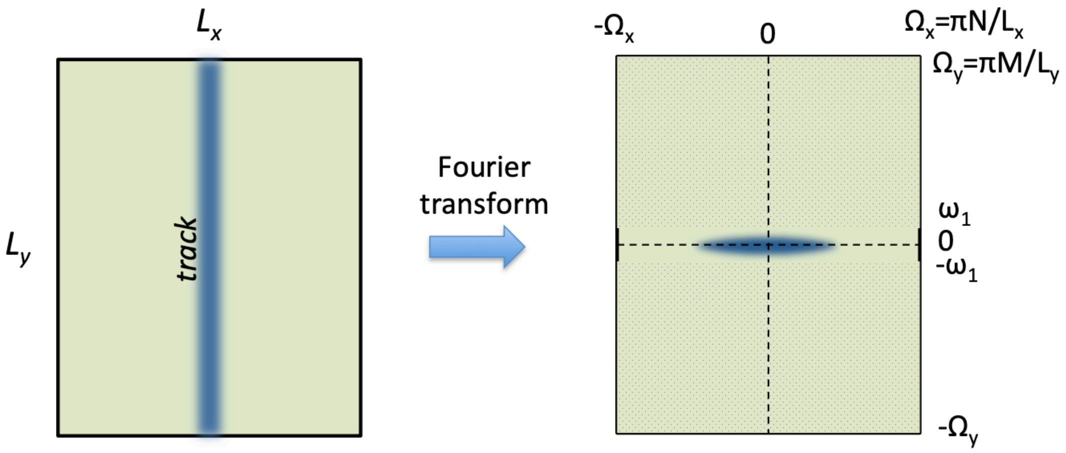

Fig.S3. Scheme of the Fourier transform of the track phase image.

The phase image of a track can be divided into a regular component $\varphi_0(x,y)$, belonging to an ideal desirable track, and a "noise" component $\varphi_{ns}(x,y)$, which could be due to fluctuations in the laser beam, fundamental fluctuations of the electron plasma cloud, and inhomogeneity in the blank material:

$$\varphi(x,y) = \varphi_0(x,y) + \varphi_{ns}(x,y) \qquad \text{(S9).}$$

Then the integral of RMS phase fluctuations across the track image can serve as a criterion of the overall track roughness:

$$R = \frac{1}{L_x L_y}\sqrt{\iint_{0,0}^{L_x,L_y} |\varphi_{ns}(x,y)|^2 dxdy} \qquad \text{(S10),}$$

that is in digital form, suitable for math treat of experimental data:

$$R = \frac{1}{NM}\sqrt{\sum_{n=1}^{N}\sum_{m=1}^{M}\left|\varphi_{n,m}^{ns}\right|^2} \qquad \text{(S11)}$$

One can turn to the Fourier space according to Parseval's theorem:

$$R = \frac{1}{NM}\sqrt{\sum_{n=1}^{N}\sum_{m=1}^{M}\left|DFT(\varphi^{ns})_{n,m}\right|^2} \qquad \text{(S12),}$$

where $DFT(\varphi^{ns})$ is the digital Fourier transform of phase fluctuation $\varphi_{n,m}^{ns}$:

$$DFT(\varphi_{n,m}^{ns})_{k,p} = \sum_{n=1}^{N}\sum_{m=1}^{M}\varphi_{n,m}^{ns}\, e^{-2\pi i\left[\frac{(n-1)(k-1)}{N}+\frac{(m-1)(p-1)}{M}\right]} \qquad \text{(S13)}$$

In reality we can only get the Fourier transform from the total phase $\varphi(x,y)$, which is measured. Then we assume that images of the regular component and the noise component are separated in the Fourier space, and the noise Fourier component $\varphi_{ns}(x,y)$ determined by the laser writing is located predominantly in the low and upper half-planes $\omega_y < -\omega_1$ and $\omega_y > \omega_1$, where ω_1 is some cut-off frequency, for example, it could be the half-width of the wait function used in the Fourier transform, that defines the width of the Fourier transform of an ideal track along y-axis (Fig.S3). Thus, (S12) is transformed to an equation, taking into account that $\varphi(x,y)$ is a real function:

$$R = \frac{2}{NM}\sqrt{\sum_{n=1}^{N/2}\sum_{m=H}^{M/2}\left|DFT(\varphi)_{n,m}\right|^2} \qquad \text{(S14)},$$

where N is the number of pixels in this image by abscissa (across a track), M is the number of pixels by ordinate (along a track), $H = \omega_1 L_y/\pi$. In our treatment of the experimental data according to formula (S14) we used the cut-off frequency ω_1 that is slightly higher than the half-width of the wait function in order to reject low frequency phase noise determined by inhomogeneity in a pristine crystal and measuring apparatus. We used H=15, while the dimension of the treated area in the crystal NxM=80x124, and the image pixel size is equalled to 0.107 μm.
